# Epidemiology of Pediatric Surgical Conditions Observed in a First-Level Hospital in Burundi

**DOI:** 10.3389/fped.2021.681478

**Published:** 2021-05-28

**Authors:** Marianna Gortan, Paola Caravaggi, Giulia Brooks, Jean Marie Vianney Butoyi, Sylvestre Bambara, Joel Nkurunziza, Mimico Mulemangabo, Gordien Nzeyimana, Protais Harakaneza, Mwajuma Nshimirimana, Costanza Tognon, Piergiorgio Gamba, Gian Battista Parigi, Daniela Dalla Gasperina

**Affiliations:** ^1^Pediatric Surgery Unit, Women's and Children's Health Department, University of Padua, Padua, Italy; ^2^Mutoyi Hospital, Mutoyi, Burundi; ^3^Department of Anesthesia and Intensive Care, University Hospital, Padua, Italy; ^4^Department of Pediatric Surgery, University of Pavia, Pavia, Italy; ^5^Department of Medicine and Surgery, University of Insubria, Varese, Italy

**Keywords:** pediatric surgery, global health, epidemiology, low-and middle-income countries, Sub-Saharan Africa, non-communicable diseases

## Abstract

**Background:** Little is known about the surgical conditions affecting the pediatric population in low-income countries. In this article we describe the epidemiology of pediatric surgical diseases observed in Mutoyi hospital, a first-level hospital in Burundi.

**Methods and Findings:** We retrospectively reviewed the records of all children (0–14 years) admitted to the Surgery ward from January 2017 to December 2017. We also reviewed the records of all the patients admitted to the Neonatology ward in 2017 and among them we selected the ones in which a surgical diagnosis was present. Five hundred twenty-eight children were admitted to the surgical ward during the study period. The most common conditions requiring hospitalization were abscesses (29.09%), fractures (13.59%), osteomyelitis (9.76%), burns (5.40%) and head injuries (4.36%). The average length of stay was 16 days. Fifty-six newborns were admitted to the Neonatology ward for a surgical condition; 29% of them had an abscess.

**Conclusions:** Conditions requiring surgical care are frequent in Burundian children and have a completely different spectrum from the western ones. This is due on one side to an under-diagnosis of certain conditions caused by the lack of diagnostic tools and on the other to the living conditions of the population. This difference should lead to intervention plans tailored on the actual necessities of the country and not on the western ones.

## Introduction

Little is known about the surgical conditions affecting the pediatric population in low-income countries (1, 2). Nevertheless, the more studies are published the more it becomes clear that pediatric surgical conditions are common in these countries and are often not treated adequately, causing premature deaths and disability (3). It is estimated that 1.7 billion children lack access to surgical care in Low and Middle Income Countries (LMICs) (4). *Bickler* in the 90s and *The Lancet Commission on Global Surgery* more recently have asserted that data assessing the actual need of surgical care in low-income countries are urgently needed in order to be able to properly take action (3, 5, 6).

For what concerns Burundi, a research on PubMed found no data about the spectrum of the surgical conditions affecting children living in the country, or about the burden of these conditions on the Burundian health system. In this article we describe the epidemiology of pediatric surgical diseases observed in Mutoyi hospital, a first-level hospital in Burundi. Moreover, data about the catchment area of the hospital, length of stay, inpatient mortality and the surgical care provided will be presented.

## Materials and Methods

### Background

Burundi is a small country (27,830 km^2^) located in eastern Sub-Saharan Africa. It is one of the most densely populated countries in Africa (11,865,821 inhabitants - July 2020 est. - 426 inhabitants/km^2^ - World Factbook) and one of the poorest countries in the world; in 2014 Burundi had the fourth highest rate of extreme poverty worldwide, with 72.9% of the population living below the international poverty line of US$1.90 per day (7, 8).

The country is now living an important population growth (annual growth: 3.18%) (7, 9). About half of the population is under-18 and this age group is projected to increase by 107% from 2015 to 2050 (10). The neonatal and under-five mortality rates are 24 and 72 per 1,000, respectively (2016) (11).

There is an important paucity of healthcare workers, with 1 medical doctor every 21,035 inhabitants, and an even lower surgeons-per-population ratio with 0.18 surgeons per 100,000, a ratio which sharply differs from the target set by the Lancet Commission Global Surgery 2030 of 40 surgeons for 100,000 inhabitants (12–14). Moreover, anesthesiologists—key figures in performing safe surgery—are almost inexistent in the Country. A *WFSA* (World Federation of Societies of Anesthesiologists) survey conducted in 2015/2016 found that there were only 6 physicians with anesthetic qualification in the country, meaning 0.06 anesthesiologists per 100,000 inhabitants (WFSA, s.d.). Anesthesia is managed by nurses with different levels of specialization in the field, as in most African countries.

Mutoyi Hospital is a first level hospital located in the province of Gitega. The hospital is equipped with a total of 317 beds. Children with surgical conditions are hospitalized in the Surgery ward (79 beds) or in the Neonatology unit (30 beds), depending on the age.

### Data Collection

We retrospectively reviewed the records of all children (0–14 years) admitted to the Surgery ward from January 2017 to December 2017. For each admission we recorded medical record number, patient's name, dates of admission and discharge, sex, age, age group (An: 0–1 months, A: 1–12 months, B: 13–59 months, C:5–9 years, D:10–14 years), area of residence, type of admission (direct, transfer, scheduled hospitalization), principal and secondary diagnosis (with their ICD-9-CM associated code), procedures performed and outcome.

We also reviewed the records of all patients admitted to the Neonatology ward in 2017 and among them we selected the ones in which a surgical diagnosis was present. On top of the data listed above, for this group of patients place of birth and modality of delivery were also recorded.

### Statistical Analysis

Data analysis was performed using SPSS version 19; SPSS Inc., Chicago, IL, USA. Categorical variables were expressed as absolute numbers and their relative frequencies; quantitative variables were presented as mean, median and range. The independent samples *t*-test was used to compare two population means and Pearson chi-square and Fischer exact test were used to compare categorical variables. A *P*-value < 0.05 was considered significant.

## Results

In 2017, 528 children were admitted to the surgical ward. The total admissions were 563 (30 children were admitted twice, 4 children were admitted three times and 1 child was admitted four times). Therefore, the readmission rate was 6%.

**Figure 1:**
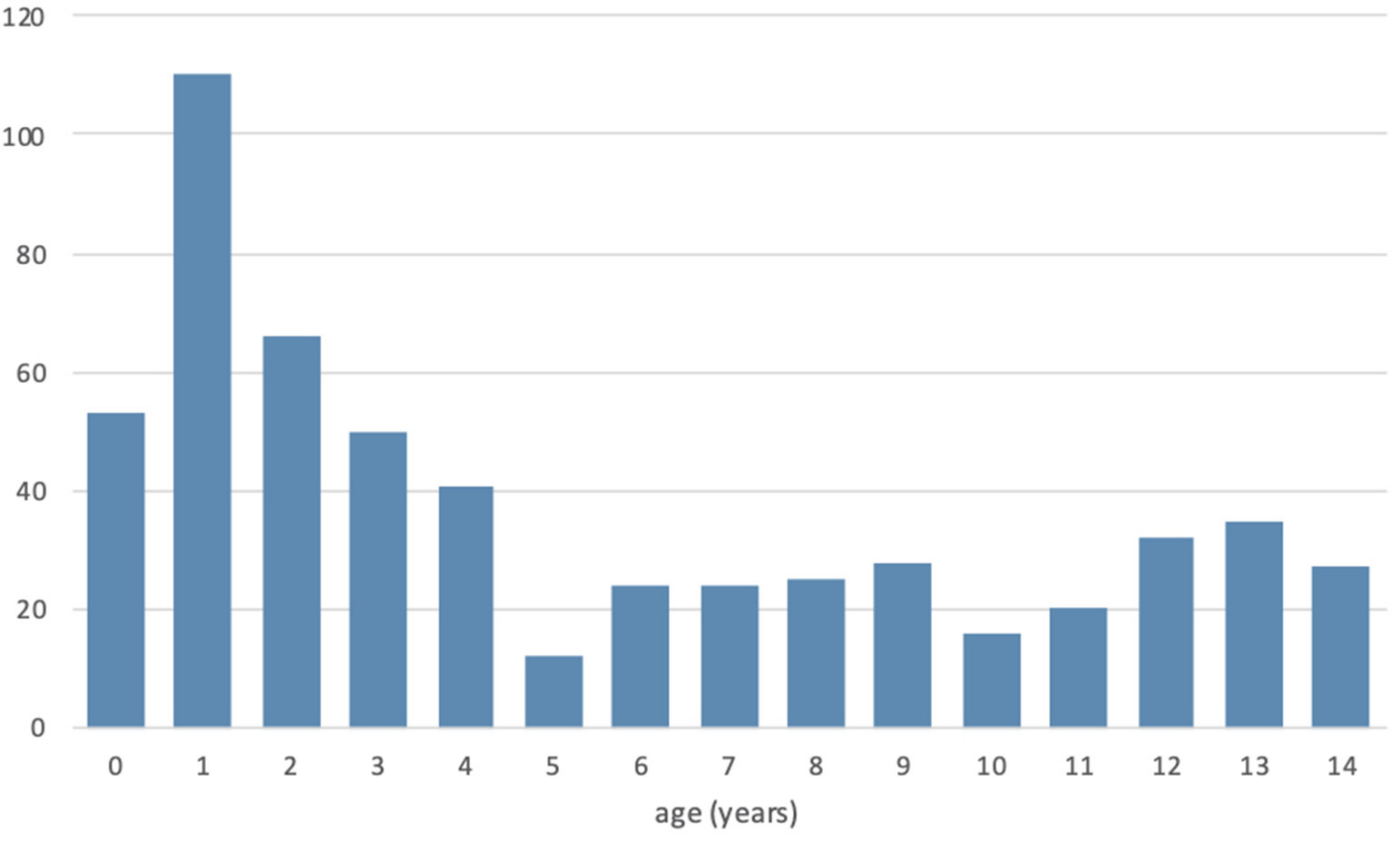
Age distribution of pediatric surgical admissions in Mutoyi Hospital, Burundi (2017).

The age distribution is shown in Figure 1. Average patients' age was 5.2 years [95% confidence interval (CI) = 5.29 ± 0.38]. 56.84% of the children were <5 years old. Average age was significantly higher in the male group than in the female one [6 vs. 4.2 years (*p* < 0.001)].

Table 1 shows the diagnoses made and their frequency. In 8 cases two major diagnoses were made, therefore the total number was 571. The most common conditions requiring hospitalization were abscesses (29.09%), fractures (13.59%), osteomyelitis (9.76%), burns (5.40%) and head injuries (4.36%).

**Table 1:** Diagnoses made for the children admitted to Mutoyi Hospital in the Surgery Ward in 2017.

**Diagnosis**	**No of admissions** **(%)**	**Sex**	
		**M (%)**	**F (%)**
**Surgical infections**	296 (51.8)	157 (53.0)	139 (47.0)
Abscess	167 (29.2)	81 (48.5)	86 (51.5)
Osteomyelitis	56 (9.8)	36 (64.3)	20 (35.7)
Pyomyositis + necrotizing fasciitis	30 (5.2)	16 (53.3)	14 (46.7)
*Tinea capitis* with bacterial superinfection	12 (2.1)	5 (41.7)	7 (58.3)
Cellulitis	11 (1.9)	6 (54.5)	5 (45.5)
Infected ulcer	10 (1.8)	8 (80.0)	2 (20.0)
Gangrene	4 (0.7)	1 (25.0)	3 (75.0)
Scabies with bacterial superinfection	3 (0.5)	2 (66.7)	1 (33.3)
Noma	1 (0.2)	1 (100)	0
Septic arthritis	1 (0.2)	0	1 (100)
Other	1 (0.2)	1 (100)	0
**Trauma and burns**	172 (30.1)	109 (63.4)	63 (36.6)
***Trauma***	*140 (24.5)*	*90 (64.3)*	*50 (35.7)*
Fracture	76 (13.3)	44 (57.9)	32 (42.1)
Head injury	25 (4.4)	17 (68,0)	8 (32.0)
Soft tissue injury	22 (3.9)	16 (72.7)	6 (27.3)
Ocular trauma	5 (0.9)	4 (80)	1 (20)
Abdominal trauma	5 (0.9)	5 (100)	0
Traumatic dislocation	3 (0.5)	2 (66.7)	1 (33.3)
Traumatic amputation	2 (0.4)	1 (50)	1 (50)
Spinal trauma	1 (0.2)	1 (100)	0
Contusion	1 (0.2)	0	1 (100)
***Burns***	*32 (5.6)*	*19 (59.4)*	*13 (40.6)*
Burn	31 (5.4)	18 (58.1)	13 (41.9)
Caustic burn of the mouth	1 (0.2)	1 (100)	0
**Congenital anomalies**	29 (5.1)	18 (62.1)	11 (37.9)
Anorectal anomaly	12 (2.1)	3 (25)	9 (75)
Uncomplicated inguinal hernia	5 (0.9)	5 (100)	0
Hydrocele	3 (0.5)	3 (100)	–
Undescended testicle	2(0.4)	2 (100)	–
Hirschsprung's disease	2 (0.4)	2 (100)	0
Spermatic cord cyst	2 (0.4)	2 (100)	–
Umbilical hernia	1 (0.2)	1 (100)	0
Pre-auricular fistula	1 (0.2)	0	1 (100)
Osteoarticular malformation of the phalanx	1 (0.2)	0	1 (100)
**Non-traumatic acute abdomen**	22 (3.9)	15 (68.2)	7 (31.8)
Complicated inguinal hernia	5 (0.9)	4 (80)	1 (20)
Intussusception	5 (0.9)	5 (100)	0
Small bowel occlusion	2 (0.4)	2 (100)	0
Necrotizing enterocolitis	1 (0.2)	1 (100)	0
Other	9 (1.2)	3 (33.3)	6 (66.6)
**Neoplasms**	15 (2.6)	10 (66.7)	5 (33.3)
Benign	11 (1.9)	6 (58.3)	5 (41.7)
Malignant^a^	4 (0.7)	4 (100)	0
**Non-traumatic orthopedic conditions**	8 (1.4)	5 (62.5)	3 (37.5)
Pathological dislocation of hip	6 (1.0)	4 (66.7)	2 (33.3)
Hip osteoarthritis	1 (0.2)	1 (100)	0
Epiphysiolysis of the hip	1 (0.2)	0	1 (100)
**Other gastrointestinal conditions**	5 (0.9)	4 (80.0)	1 (20.0)
Rectal prolapse	2 (0.4)	1 (50)	1 (50)
Foreign body in the esophagus	1 (0.2)	1 (100)	0
Pyloric stenosis	1 (0.2)	1 (100)	0
Other	1 (0.2)	1 (100)	0
**Urology**	5 (0.9)	4 (80.0)	1 (20.0)
Orchi-epididymitis	3 (0.5)	3 (100)	–
Urethral caruncle	1 (0.2)	0	1 (100)
Bladder stone	1 (0.2)	1 (100)	0
**Iatrogenic pathologies**	5 (0.9)	5 (100)	0
Abdominal fistula	2 (0.4)	2 (100)	0
Compartment syndrome	2 (0.4)	2 (100)	0
Disruption of wound	1 (0.2)	1 (100)	0
**Plastic surgery conditions**	5 (0.9)	4 (80)	1 (20)
Contracture scar	4 (0.7)	3 (75)	1 (25)
Keloid scar	1 (0.2)	1 (100)	0
**Other**	9 (1.6)	5 (55.5)	4 (44.5)
Snake bite	2 (0.4)	0	2 (100)
Dog bite	1 (0.2)	1 (100)	0
Drowning	1 (0.2)	0	1 (100)
Lightening electrocution	1 (0.2)	1 (100)	0
Other	4 (0.8)	3 (75)	1 (25)
Total	571 (100)	336 (58.8)	235 (41.2)

a *Distinction based on clinical characteristics and evolution of the neoplasms*.

Concerning the type of admission, 67.9% of patients were admitted directly to the hospital with an emergent condition, 9.8% had a scheduled hospitalization, 8.9% were transferred from other hospitals, 8% were transferred from *Centres des Santé* (*CdS –* primary health care centers where basic health care is provided), 4.6% were transferred from the Pediatric ward of the hospital, 0.9% came to the hospital for a routine check-up but their conditions required hospitalization.

**Figure 2:**
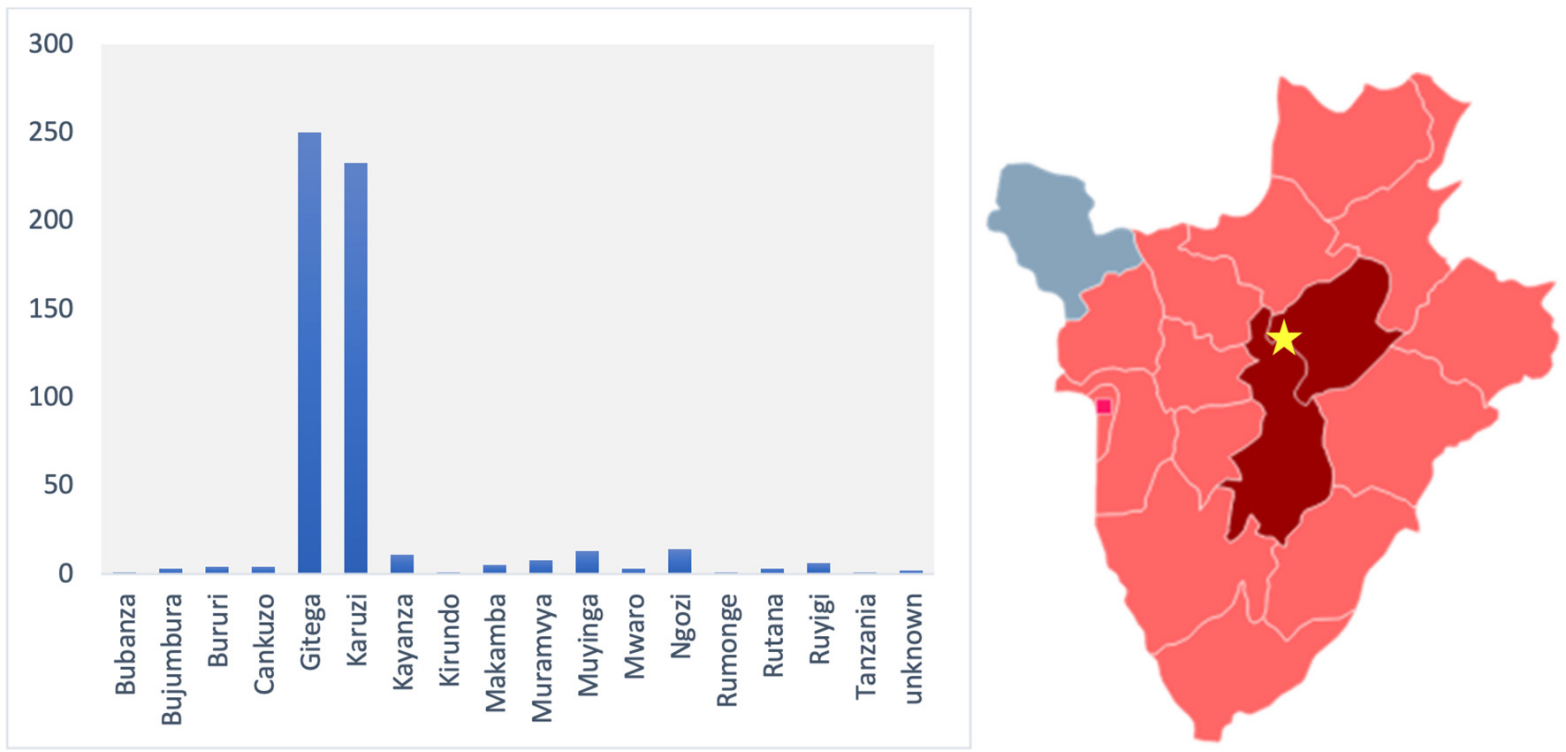
Provinces from which children traveled to Mutoyi hospital in 2017. The location of Mutoyi Hospital is marked by a star in the map. The provinces of Gitega and Karuzi, from which the majority of patients came from, are colored in red. The provinces from which at least 1 patient came from are colored in pink.

Most patients (85.8%) lived in the provinces of Gitega and Karuzi (Figure 2), but patients came from throughout the country. Patients who were not from the provinces of Gitega or Karuzi came to the hospital mainly for Fractures (22.5%), Osteomyelitis (22.5%), Anorectal malformations (10%), Abscesses (8.8%) and Neoplasms (6.3%).

The average length of stay was 16 days (95% CI = 16.37 ± 2.37, range: 1–372 days).

A total of 417 operations were performed in the study period; 178 patients did not undergo any operation during their hospitalization, 12 patients underwent 2 operations, 7 patients underwent 3 operations and 1 patient underwent 7 operations within a single hospitalization. Among those who underwent more than one operation, 8 children were affected by osteomyelitis or myositis.

A total of 14 children died while in hospital, resulting in an overall mortality of 2.7%: 6 children died of surgical infection sequelae (4 of necrotizing fasciitis, 2 of osteomyelitis of the occipital bone, 1 of submandibular abscess); 3 children died of head trauma; 1 child died of respiratory distress right after a surgical operation for a colic perforation; 1 child died the first day after surgery for what was intraoperatively interpreted as Hirschsprung disease (no service of Pathological Anatomy is present in Mutoyi hospital); 1 child died of respiratory distress in the context of intestinal occlusion before reaching the OR; 1 child died of necrotizing enterocolitis and 1 child died of malaria during hospitalization for a massive burn. The average age of children who died was 2.57 years, significantly lower (*p* < 0.001) than the overall one.

### Surgical Infections

Abscesses were responsible for the largest number of admissions. The average age of children with abscesses was significantly lower than the overall average age [2.56 vs. 5.2 (CI 95%: 2.56 ± 0.52, range: 0–14 years)]. The average length of stay was 6.5 days (CI 95%: 6.5 ± 0.58, range: 1–34 days), significantly shorter than the overall average length of stay. The most common locations of abscesses were the neck (28.4%), the face (18.3%) and the inferior limbs (23.7%). The average interval between the onset of the symptoms and the admission to the hospital was 7.58 days (CI 95%: 7.58 ± 0.79, range: 1–35 days). In 149 cases a procedure of incision and drainage was performed, whereas 18 cases were treated just medically, mainly with Cloxacillin orally.

Osteomyelitis was the third cause of admission (after abscesses and fractures). Average age of children with osteomyelitis was 9.7 years (CI 95%: 9.7 ± 0.98, range: 0–14 years), significantly higher (*p* < 0.001) than the overall average age. Average length of stay was 46 days (CI 95%: 46 ± 15.2, range: 1–372 days), significantly longer than the overall one. The most common localizations were the tibia (44%) and the femur (20%). In 47% of the admissions for osteomyelitis a procedure of sequestrectomy was performed.

### Trauma and Burns

Seventy-six children were hospitalized for a fracture. Average age in this diagnostic group was 7.37 years (CI 95%: 7.37 ± 0.9, range: 0–14 years), significantly higher than the overall average age (*p* < 0.001). Average length of stay was 18 days (CI 95% 18 ± 3.12, range: 1–54 days). The most common fracture which required a hospitalization was femur fracture (47%). Children with femur fractures were mainly hospitalized because a skeletal traction was indicated: the traction was applied for 25.8 days on average (CI 95%: 25.8 ± 2.15). The second most common fracture was the humeral fracture (23%); also in this case the hospitalization was mainly due to the necessity of a skeletal traction (66.6%), which was kept in place for 5.8 days on average (CI 95%: 5.8 ± 2.1). In 17.1% of cases there was a fracture of the forearm and in 15.8% of cases there was a fracture of the leg; in these children, the hospitalization was mainly due to the necessity of decreasing the edema before putting a cast. 18.42% of the fractures which required hospitalization were caused by a fall from a tree and another 25% were caused by traffic accidents, that were also responsible for 64% of head traumas.

Thirty-two children were hospitalized for burns, among whom 1 presented a caustic burn of the mouth. The average age was 3.81 years (CI 95%: 3.81 ± 1.42: range: 0–14 years). The average length of stay was 26.19 days (CI 95%: 26.19 ± 15.85; range: 2–204 days, where “204” is an approximation as this patient was still hospitalized at the end of data collection). 35.48% of the burns were caused by an accidental fall in the domestic brazier.

### Congenital Anomalies

Anorectal malformations (ARMs) were the most common congenital anomalies requiring hospitalization in order to be surgically treated. All the children of this group had a colostomy performed at birth; in 2 cases a posterior sagittal anorectoplasty was performed (average age: 19 months), whereas 7 children were hospitalized for scheduled intestinal recanalization (average age: 20 months). In three cases a fistula was present, in 3 cases no fistula was found, whereas in 2 cases no details about the presence of a fistula were given.

### Neonatology

In 2017, 1,840 newborns were hospitalized in the Neonatology department: among them only 56 had a surgical condition. Table 2 summarizes the diagnosis made and their frequency. Age at hospitalization was 14 days on average (CI 95%: 14 ± 4.13; range: 0–81 days). Average length of stay was 13 days (CI 95%: 13 ± 3; range: 0–62 days). A surgical procedure was performed in just over the half of the cases. 10.7 % were transferred from a *Centre de Santé*, 5.4% were transferred from another hospital. Seven patients out of 56 died while in hospital; 2 presented an Anorectal Malformation and were transferred from other centers at 2 and 3 days of life, respectively, in compromised conditions: one died before reaching the operating room, while the other died the third postoperative day; 2 were born with multiple malformations; 1 presented an omphalocele and died of sudden cardiac arrest; 1 had an intestinal occlusion and died before reaching the operative room; 1 presented with an abscess of the inferior limb and died for advanced sepsis.

**Table 2:** Surgical diagnosis made in the Neonatology Department of Mutoyi Hospital (2017).

**Diagnosis**	**No of admissions (%)**	**F (%)**	**M (%)**
Abscess	29 (51.79)	19 (65.5)	10 (34.5)
Cleft lip/palate	6 (10.72)	3 (50)	3 (50)
Anorectal malformation	5 (8.93)	1 (20)	4 (80)
Omphalocele	3 (5.36)	1 (33.3)	2 (66.7)
Multiple malformations	2 (3.58)	1 (50)	1 (50)
Intestinal occlusion	2 (3.58)	1 (50)	1 (50)
Clubfoot	2 (3.58)	0 (0)	2 (100)
Umbilical hernia	1 (1.79)	1 (100)	0 (0)
Femur fracture	1 (1.79)	1 (100)	0 (0)
Humeral fracture	1 (1.79)	1 (100)	0 (0)
Pyloric stenosis	1 (1.79)	0 (0)	1 (100)
Skull malformation	1 (1.79)	1 (100)	0 (0)
Limb malformation nos	1 (1.79)	1 (100)	0 (0)
Intestinal perforation	1 (1.79)	1 (100)	0 (0)
Total	56 (100)	32 (57.1)	24 (42.9)

Abscesses were in 86% of cases localized at the breast, so they were probably secondary to a neonatal mastitis. The Female-to-Male ratio for the breast abscesses was 2:1, consistent with the published literature (15). A surgical procedure was performed in 75.6% of patients with abscesses, whereas the remaining patients were treated just with antibiotics, which were usually intravenous Ampicillin, associated to intravenous Gentamicin in severe cases.

Patients with cleft lip/palate were hospitalized to monitor their nutrition in their first days of life. In the case of simple cleft lip anomalies surgical interventions were performed.

The 3 patients with Anorectal malformations who survived had in 2 cases a high ARM, so a colostomy was performed, while in 1 case the ARM was low and so it was possible to perform a corrective treatment.

## Discussion

This study offers insights about the pediatric surgical conditions which can most commonly be found in a first-level Burundian hospital. Mutoyi's epidemiology can be considered quite representative of the surgical conditions which affect the Burundian pediatric population, considering its extended catchment area. However, it is important to highlight that Mutoyi hospital represents an exception in terms of variety and quality of medical services performed, as most of first-level Burundian hospitals completely lack surgical services.

The average age of the hospitalized patients was similar to those of other similar studies conducted in African countries (2, 16). Nevertheless, our data could have been influenced by the fact that in Burundi health care is free until the age of five and so parents often lie about the actual age of their children in order to get free care. Considering this bias, the actual average age could have been a little higher than the one calculated.

The overall average length of stay (LOS) was higher than the average LOS of western pediatric surgery departments; for example, in 2014 in Lombardy, a region of northern Italy, the average LOS for Pediatric surgery departments was 3.4 days (17), greatly inferior to the one observed in this study. This is due to the profoundly different spectrum of diseases but also to the difficulties of having an outpatient follow-up (considering the long distances people usually have to cover before reaching the hospital and the scarce hygienic conditions) which imply the need for prolonged hospitalizations in order to achieve a complete recovery.

Almost two thirds of patients were admitted to the hospital for an emergent condition; this data sharply differ from west-world ones, where most patients are hospitalized in elective regimen.

Infections requiring surgical treatment were the main cause of hospitalization. This fact is clearly related to the poor hygienic conditions in which the majority of the population lives in and to the important delay that normally elapses between the onset of the pathology and the arrival to a health care facility. This entails that conditions that could have been treated just medically in an early state become of surgical pertinence.

Traumas were the second major diagnostic group. A non-negligible number of traumas was caused by traffic accidents, which nowadays represent a great problem for low-income countries, where the increasing availability of vehicles is not matching with an improvement in the security of the road network. Another interesting cause of traumas was the fall from trees. Falls from trees did not have an important seasonal variation, as noticed in other studies done in African countries (2).

Burns were the third major diagnostic group. Even in this case, no seasonal variations were noticed. The average age of children with burns was lower than the overall one. This is probably related to the immature proprioception and the lack of sense of danger of younger children. Concerning older children, extensive burns caused by fire were seen in patients who were referred to have had seizures in the proximity of the domestic fireplace. In our hospital experience, seizures are in certain cases due to an underlying epileptic disease (and in these cases it is not rare to see recurrent burns), but more commonly they are observed during malaria febrile peaks.

Anorectal malformations were the most common congenital malformations observed. This data differs from the European ones, where other types of congenital anomalies have a higher incidence. This difference is clearly due to the fact that anorectal malformations can be easily diagnosed with a simple inspection, while other types of anomalies (like heart defects, esophageal atresia and so on) are far more difficult to diagnose, leading to neonatal deaths from undetermined causes.

One of the limits of this study is its retrospective nature. Nonetheless, records were accurately filled in and all information needed was found. Moreover, we think a prospective study would not bring to a better diagnostic classification until more advanced diagnostic tools will be present at the hospital.

As said before, Burundi is living an important population growth, which will lead to a greater number of children in the next years. Surgical conditions in this part of the population are frequent and have a completely different spectrum from the western ones. This is due on one side to an under-diagnosis of certain conditions caused by the lack of diagnostic tools and on the other to the living conditions of the population. This difference should lead to intervention plans tailored on the actual necessities of the country and not on the western ones: as pointed out by doctor Laura Farmer in her Presidential address given at the 48th Annual meeting of APSA (American Pediatric Surgical Association), lots of well-intentioned initiatives to empower children's surgical care in low-income countries fail as they are directed by experts who operate in high-income countries and do not know which are the actual needs of Low-income countries (18). In our study, infections requiring surgical treatment, traumas and burns were the conditions which more frequently required hospitalization. This data suggests that intervention plans should include strong prevention protocols, designed to improve hygienic conditions and to raise awareness on the need of prompt medical evaluation. Our study could be a useful tool for governmental and non-governmental institutions to implement these prevention protocols.

Surgical skills are badly needed, and they need to be taught to a wider portion of health care workers in order to fulfill the urgent request, as pointed out by other authors (3, 19). In Mutoyi in 2017 there was just one surgeon specialized in Gynecology and Obstetrics with experience in General Surgery. Nevertheless, a large amount of different surgical pathologies was treated, and this thanks to a stimulating teaching environment. Also, some of the nurses were empowered to do some simple procedures on their own. Specialization in Burundi costs a lot of money and specialized surgeons usually work in the capital, so we cannot look only for specialized surgeons to cope with the surgical needs, but we need to involve a larger portion of health care workers.

Another critical area in which advances are urgently needed is anesthesia: no safe surgery can be performed without safe anesthesia. In Mutoyi Hospital no anesthesiologists were present and anesthesia was carried out by few nurses who were taught anesthesia basics in seminars hold by specialists from high-income countries, as often happens in third-world countries. It is clear, then, that these nurses could not have the medical knowledge to safely cope with situations just beyond ordinary or with delicate patients, as children often are. Moreover, these nurses had a very limited range of instruments and drugs to work with, making their job even harder. Once more, it is critical to improve specialized training in the first place. It is clear that it would be a waste to provide more sophisticated instruments and drugs to anesthesia providers without investing in their education.

Finally, we want to emphasize the fact that this is one of the few studies conducted in a first-level hospital in LMICs. Most of epidemiological studies published in recent years on the subject were conducted in third level hospitals, depicting, as expected, a different incidence of disease groups. For example, in a study conducted in a third-level center in Uganda, congenital anomalies were the first diagnostic group, followed by trauma, infections and tumors (20). These data are surely precious, but we need to consider that in LMICs only a very little part of the population has access to third-level hospitals due to the costs and to the distances that people need to cover before reaching more advanced care. We think that more initiatives should address first-level hospitals in order to reach as many children as possible.

## Data Availability Statement

The original contributions presented in the study are included in the article/supplementary material, further inquiries can be directed to the corresponding author/s.

## Author Contributions

MG, PC, GP, and DD contributed to conception and design of the study. PC, JB, SB, JN, MM, GN, PH, and MN acquired the data. MG organized the database and wrote the first draft of the manuscript. DD performed the statistical analysis. PC, GB, CT, PG, GP, and DD wrote sections of the manuscript. All authors contributed to manuscript revision, read, and approved the submitted version.

## Conflict of Interest

The authors declare that the research was conducted in the absence of any commercial or financial relationships that could be construed as a potential conflict of interest.

## References

[1] Butler EK Tran TM Nagarajan N Canner J Fuller AT Kushner A. Epidemiology of pediatric surgical needs in low-income countries. PLoS ONE. (2017) 12:e0170968. 10.1371/journal.pone.0170968 28257418 PMC5336197

[2] Bickler SW Sanno-Duanda B. Epidemiology of paediatric surgical admissions to a government referral hospital in the Gambia. Bull World Health Organ. (2000) 78:1330–6. 10.1590/S0042-96862000001100008 11143193 PMC2560634

[3] Bickler SW Kyambi J Rode H. Pediatric surgery in sub-Saharan Africa. Pediatr Surg Int. (2001) 17:442–7. 10.1007/s003830000516 11527185

[4] Mullapudi B Grabski D Ameh E Ozgediz D Thangarajah H Kling K. Estimates of number of children and adolescents without access to surgical care. Bull World Health Organ. (2019) 97:254–8. 10.2471/BLT.18.216028 30940982 PMC6438256

[5] Holmer H Lantz A Kunjumen T Finlayson S Hoyler M Siyam A. Global distribution of surgeons, anaesthesiologists, and obstetricians. Lancet Glob Health. (2015) 3 (Suppl. 2):S9–11. 10.1016/S2214-109X(14)70349-3 25926323

[6] Meara JG Leather AJ Hagander L Alkire BC Alonso N Ameh EA. Global surgery 2030: evidence and solutions for achieving health, welfare, and economic development. Lancet. (2015) 386:569–624. 10.1016/S0140-6736(15)60160-X 25924834

[7] The World Bank. Republic of Burundi Addressing Fragility and Demographic Challenges to Reduce Poverty and Boost Sustainable GrowthSystematic Country Diagnostic. Washington, DC: The World Bank (2018).

[8] The World Bank. République du Burundi Évaluation de la pauvreté au Burundi. Washington, DC: The World Bank Group Publications. (2016).

[9] Bevir M Hurt SR. World Development Indicators. In Encyclopedia of Governance. Thousand Oak, CA: SAGE Publications, Inc. (2012).

[10] UNICEF. Generation 2030 AFRICA 2.0. (2017). Available online at: http://www.unicef.org/publications/files/Generation_2030_Africa.pdf (accessed March 2021).

[11] UNICEF. Multiple Indicator Cluster Surveys (MICS). (2018). p. 1–11. Available online at: https://data.unicef.org/resources/resource-type/datasets/ (accessed March 2021).

[12] Ministère de la Santé Publique et de la Lutte contre le SIDA (Burundi). Annuaire Statistique Sanitaire. (2016). Available online at: http://minisante.bi/wpcontent/uploads/annuaires_statistiques/annuaire%20statistique%202016.pdf (accessed March 2021).

[13] World Health Organization. Stratégie de coopération de l'OMS avec le Burundi, 2016-2018. (2017). Available online at: https://apps.who.int/iris/handle/10665/272368 (accessed March 2021).

[14] O'Flynn E Andrew J Hutch A Kelly C Jani P Kakande I. The specialist surgeon workforce in East, Central and Southern Africa: a situation analysis. World J Surg. (2016) 40:2620–7. 10.1007/s00268-016-3601-3 27283189

[15] Masoodi T Mufti GN Bhat JI Lone R Arshi S Ahmad SK. Neonatal mastitis: a clinico-microbiological study. J Neonatal Surg. (2014) 3:2. 10.47338/jns.v3.66 26023473 PMC4420425

[16] Manickchund Y Hadley GP. Paediatric surgery outreach: analysis of referrals to a tertiary paediatric surgery service to plan an outreach programme Kwa-Zulu Natal, South Africa. Trop Doct. (2017) 47:305–311. 10.1177/0049475517718103 28682220

[17] INSTAT. Regional Statistical Yearbook 2014. (2014). Available online at: https://www.asr-lombardia.it/asrlomb/en/pubblicazioni (accessed March 2021).

[18] Farmer DL. Audacious goals - 2.0 the global initiative for children's surgery. J Pediatr Surg. (2017) 53:2–11. 10.1016/j.jpedsurg.2017.10.007 29173774

[19] Mock CN Donkor P Gawande A Jamison DT Kruk ME Debas HT. Essential surgery: key messages from disease control priorities, 3rd edition. Lancet. (2015) 385:2209–19. 10.1016/S0140-6736(15)60091-5 25662414 PMC7004823

[20] Cheung M Kakembo N Rizgar N Grabski D Ullrich S Muzira A. Epidemiology and mortality of pediatric surgical conditions: insights from a tertiary center in Uganda. Pediatr Surg Int. (2019) 35:1279–89. 10.1007/s00383-019-04520-2 31324976

